# Effects of Reed Biochar Mass Fraction on the Properties of Polypropylene/Reed Char Composites

**DOI:** 10.3390/polym14112212

**Published:** 2022-05-30

**Authors:** Yunpeng Ye, Dongfang Zou, Shuang Si, Xingong Li

**Affiliations:** School of Materials Science and Engineering, Central South University of Forestry and Technology, Changsha 410004, China; 18057978983@163.com (Y.Y.); zdfwood2021@163.com (D.Z.); sswood2023@163.com (S.S.)

**Keywords:** polypropylene, compound material, reed charcoal, mechanical properties, thermal properties

## Abstract

Reed charcoal/polypropylene (RC/PP) composites were prepared by melt-blending and molding processes. The effects of RC addition (by mass fraction) on its mechanical properties were investigated and the mechanism characterized. The results showed that RC and PP were physically bonded and formed a mechanical interlocking matrix. The water absorption rate of these composites was <1% at 168 h. As the RC mass fraction increased, the tensile modulus, crystallinity, and energy storage modulus of the composites increased and then decreased, with the tensile modulus reaching a maximum of 679.4 MPa. The thermal decomposition rate peak and starting melt temperature increased by 14.8 and 2.5 °C, respectively, compared to pure PP, and the energy storage modulus reached a maximum of 3752.8 MPa at 40 wt% RC. The addition of RC in appropriate amounts improved the rigidity and thermal stability of these composites.

## 1. Introduction

More than one-third of the cities in China are deep in the dilemma of a garbage siege [1,2]. The proportion of waste plastic products in solid waste has reached 15–20%. The fatal drawback of plastic products is that they are difficult to degrade or even nondegradable. Waste plastic treatment currently involves three main methods, including in landfill, incineration, and recycling. In landfill treatment, plastic decay in soil is not only long-term, with some plastics unable to decay, it also affects soil permeability and damages soil quality, thus affecting plant growth. Incineration treatment is easy, but can release toxic chemicals, such as dioxins and other harmful gases, as well as a large amount of greenhouse gases. Thus, from the perspective of resource conservation, the recycling of waste plastics is the direction for development now and in the future. Compounding waste plastics with other materials to prepare composite materials is one of the important means for recycling and reusing waste plastics.

Biomass char is a porous material with strong adsorption properties [3,4]. The main raw material for the preparation of biomass char is wood. However, with the shortage of wood resources in the world, researchers are increasingly seeking other environment-friendly, renewable, and low-cost alternative resources. Biochar prepared from low-density resources, such as reeds, straws, fruit shells, and sugarcane, by pyrolysis under anaerobic or anoxic conditions at certain temperatures (<700 °C) have been found to have well-developed pore structures and high specific surface areas [5,6,7,8]. Among these, activated reed biochar is mainly composed of aromatic hydrocarbons and elemental carbon (C) or C with a graphite structure. In addition to C, char also includes hydrogen, oxygen, nitrogen, and sulfur, as well as small amounts of trace elements. Its specific surface area can reach 1395 m^2^/g [9], which is higher than the average specific surface area of biomass char. Reed is widely distributed throughout the world, but its short growth cycle and large annual production have led to a large amount of reeds not being used effectively and rotting in the ground, as well as causing ecological damage. The use of reed pyrolysis into biomass carbon to produce carbon plastic composites can broaden the scope of application of reed resources and solve the ecological problems caused by reeds. Such chars can be used as adsorbent materials, fuels, and char-based composites, and its high dispersibility and well-developed pore structures yield them easily compounded with polymers, such as plastics, to prepare char/plastic composites [10,11,12].

Charcoal/plastic composites have excellent properties, such as high strength, water resistance, no formaldehyde release, and formaldehyde absorption from the environment, and can be widely used in furniture manufacturing, interior decoration, vehicle interiors, and other fields. Researchers have performed many studies on biomass charcoal-plastic composites [13,14,15,16,17]. Zhao et al. [18] used bamboo charcoal and a polypropylene composite to prepare charcoal-plastic composites and, when the content of bamboo charcoal was 10%, the tensile strength of charcoal/plastic composites reached a maximum of 20.51 MPa, which is 12.6% higher than the tensile strength of pure polypropylene (PP). Das et al. [19] used pine biochar and PP composites to prepare charcoal/plastic composites, and their results showed that the addition of the appropriate amount of charcoal to PP improved the tensile and flexural moduli and thermal stability of these composites. Peterson [20] prepared composites with good tensile strength, elongation, and toughness using corn starch and corn stover biochar compounded with styrene-butadiene rubber. Das et al. [21] prepared biocomposites by adding pine biochar and PP to four biomass wastes, including rice husk, coffee husk, coarse wool, and landfill wood, and found that the resulting composites exhibited high tensile/bending properties along with good thermal stability. The aforementioned studies have shown that good-performance RC/plastic composites can be prepared by compounding biomass char with polymers, but there have been few studies regarding the preparation of RC/plastic composites by compounding reed biochar with polymers. In this study, reed char and PP were used as the main raw materials for preparing reed char/plastic composites by melt-blending and molding. The effects of the reed char mass fraction on the physical and mechanical properties of reed char/plastic composites were investigated, with the aim to provide theoretical support for the development of reed char-plastic composites.

## 2. Experimental Materials and Methods

### 2.1. Experimental Materials

#### 2.1.1. Raw Materials

RC, particle diameter 75 μm, was collected from Dongting Lake (Hunan, China); and PP, T30S, molecular weight of 80,000–150,000 daltons, melt flow rate of 2.5–4.5 g/10 min, and density of 0.90–0.91 g/cm^3^, was purchased from China National Petroleum Corp. (Beijing, China).

#### 2.1.2. Experimental Apparatus

A 101A series electric blast dryer (Shanghai General Factory of Experimental Instruments, Shanghai, China), Sigma 300 scanning electron microscope (Carl Zeiss AG, Oberkochen, Germany), RAffinity-1 Fourier infrared spectrometer (Shimadzu Corp., Kyoto, Japan), XK-1608 double-roller refiner (Shanghai Rubber Machinery Factory, Shanghai, China), QD86107 hot press (Suzhou Xinxiexiang Machine Manufacturing Co. Ltd., Suzhou, China), DCS-R-100 Universal Mechanical Experiment Machine (Shimadzu Corp.), 200F3 Scanning Calorimeter (Netzsch-Gerätebau GmbH, Selb, Germany), PerkinElmer STA 8000 (PerkinElmer, Inc., Waltham, MA, USA), 8411 electric vibrating sieve machine (Anhui Changhao High Pressure Pipe Fittings Co., Anhui, China), and JP-1500B High-speed universal crusher, (Jiangyin Manda Machinery Co. Ltd., Jiangyin, China) were used in this study.

### 2.2. Experimental Methods

#### 2.2.1. Raw Material Handling

RC powder was placed in the 8411 electric vibrating sieve machine to obtain 200 mesh to fine RC. The powder was then placed in a blast drying oven at 105 °C for 16 h until there was no further change in quality and then removed and placed in a self-sealing bag and set aside. PP was placed in a drying oven and dried continuously at 80 °C for 16 h, then removed and placed in a self-sealing bag and set aside.

#### 2.2.2. Preparation of RC/PP Composites

PP/RC mixes with RC mass fractions of 0, 20, 30, 40, and 50 wt% were weighed and mixed, each with a total mass of 450 g.

Mixing was carried out on the open double-roller machine at a mixing temperature of 185 °C for 10 min. After 4 h of cooling, the material was crushed to granular form by a high-speed universal crusher, poured into a hot pressing mold (200 mm × 200 mm × 5 mm), and the surface made even and flat, with a stacking height of ~1 mm above the mold. Two layers of tin foil were attached to the top and bottom surfaces to prevent the material from adhering to the hot pressing iron plate, and the mold was slowly put into the hot pressing machine for 5 min, at 185 °C and a hot pressing pressure range from 4 to 6 MPa. The hot-pressed composites were finally placed in corresponding sealed bags for subsequent testing and examination.

### 2.3. Testing and Characterization Methods

#### 2.3.1. Mechanical Properties Characterization

Tensile and bending tests and impacts of RC/PP composites with different mass fractions were carried out on the universal mechanical testing machine, with the reference standards for tensile and impact tests, including GB/T1040-2006 [22] and GB/T1043-2008 [23]. The speed was 20 mm/min, and in sample testing, at least 5 parallel samples were examined and averaged. Bending test reference standards were GB/T9341-2000 [24], with the speed at 5 mm/min, and 5 samples tested and averaged.

#### 2.3.2. Characterization of Moisture Resistance

The Reference Standard GB/T1034-2008 Method [25] for the determination of water absorption in plastics was used here. A specimen was dried in an oven at 50 °C for 24 h, cooled to room temperature in a desiccator, weighed (*M_1_*), and placed into a constant-temperature and -humidity chamber with 50% relative humidity (RH) and 23 °C. After 24 h, the specimen was reweighed (*M_2_*) within 1 min of removal from the chamber. The specimen was then placed in a 50 °C oven to dry for 24 h, after which it was cooled to room temperature in a desiccator and weighed again (*M_3_*). The water absorption mass fraction (*C*) was calculated using

(1) $$C=\frac{M_{2}-M_{3}}{M_{1}}\times 100\%$$

#### 2.3.3. Thermogravimetric Analysis (TG)

A sample was hand-sawed at the two ends (20 cm from the end) and the middle of the 100 mm long charcoal-plastic composite material. The resulting sawing particles were collected, the 50–80 mesh composite material sawdust sieved, and the resulting powder placed in the oven at 105 °C until the moisture content was <2%. Dried samples of 4–5 mg were placed in a platinum tray and the temperature increased from 3 to 850 °C at a rate of 20 °C/min. The test atmosphere was nitrogen and the mass loss of the composite and its primary differential curve were recorded, with 3 samples measured and averaged per group.

#### 2.3.4. Differential Scanning Calorimetry (DSC)

About 10 mg of RC/PP composite samples was weighed and their thermal properties measured using differential scanning calorimetry (200F3), from room temperature to 260 °C at a rate of 5 °C/min, then a constant temperature of 5 min with a nitrogen purge of 50 mL/min. The crystallinity *X_C_* was calculated using

(2) $$X_{C}\left(\%\right)=\frac{\Delta H_{m}}{\Delta H_{0}\times X_{PP}}\times 100\%$$

where Δ*H_m_* is the melt enthalpy of the RC/PP composite, Δ*H*_0_ is the melt enthalpy of 209 J/g at 100% crystallization of PP [26], and *X_PP_* is the mass fraction of PP in the RC/PP composite.

#### 2.3.5. Micromorphological Analysis (SEM)

The surface micromorphology of RC and the tensile fracture surface of RC/PP composites were observed using field emission scanning electron microscopy (SEM, Sigma 300, Oberkochen, Germany) with a gold-sputtered surface and emission voltage of 3.0 kV.

#### 2.3.6. Dynamic Thermomechanical Analysis (DMA)

Dynamic thermomechanical analysis (DMA) characterizes the dynamic modulus and mechanical loss of a material as a function of temperature or frequency under sinusoidal periodic vibratory loading. In this study, the dynamic mechanical properties of RC/PP composite specimens were tested using a dynamic mechanical properties tester (TADMA 850, TA Instruments Ltd., New Castle, DE, USA) with a length, width, and thickness of 60, 10, and 5 mm, respectively, at 100–200 °C, a vibration frequency of 1 Hz, and a temperature rise rate of 3 °C/min.

## 3. Results and Discussion

### 3.1. Analysis of the Effects of RC Content on the Elastic Modulus and Impact Strength of RC/PP Composites

**Figure 1:**
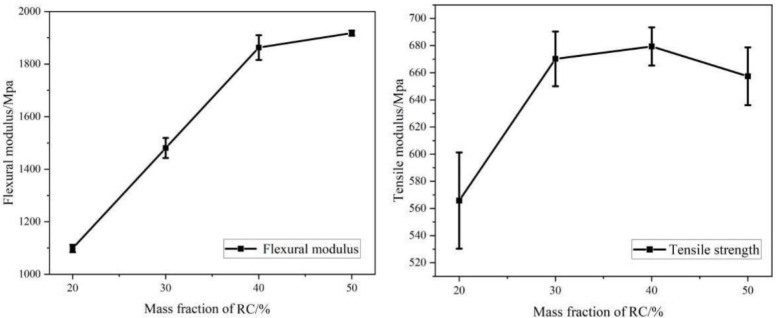
Tensile and flexural properties of RC/PP Composites.

**Figure 2:**
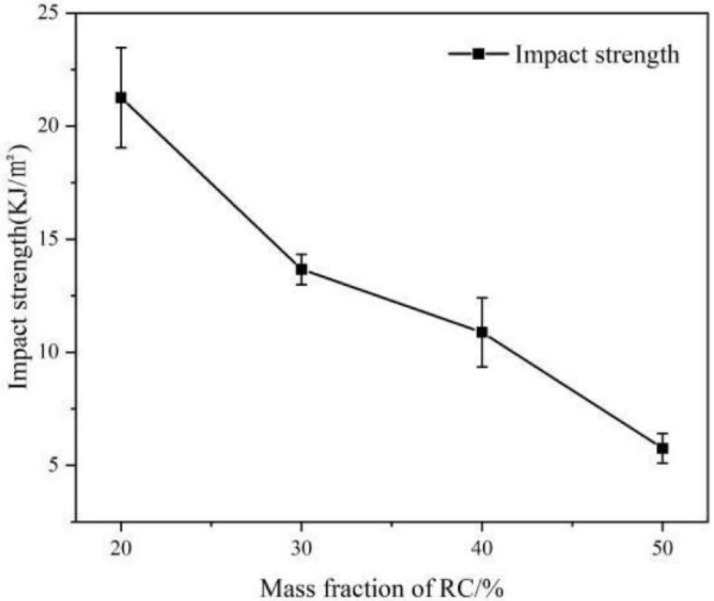
Impact properties of RC/PP Composites.

The RC/plastic ratio had a large influence on the properties of these composites during the hot compression molding process. The impact, tensile, and bending strengths of RC/PP composites made with different charcoal/plastic ratios were tested (Figure 1 and Figure 2).

Changes in the bending and tensile properties of composites with different RC contents showed that, with increased RC addition, the bending modulus gradually rose and the tensile modulus first rose and then declined. When the mass fraction of RC was 40 wt%, the bending and tensile elastic moduli of RC/PP composites reached higher values, at 1862.8 and 679.4 MPa, respectively. This increase in elastic modulus was attributed to intermolecular forces between the RC particles and PP molecules, as RC particles restricted the mobility of PP polymer chains and led to increased composite stiffness [27,28,29]. Thus, the appropriate amount of RC acted as a nucleus during PP crystallization, enabling more PP molecular chains to be arranged in a regular manner during the crystallization process. This increased PP crystallinity was manifested macroscopically by the increased stress required for the material to yield. Das et al. [30] obtained similar results, i.e., the tensile modulus of polymer/biochar composites increased with increased C content in the polymer matrix, but excess RC lacked an homogeneous dispersion due to the formation of agglomerates in the PP matrix [31]. This leads to a weakening of the mechanical interlocking effect between the RC filler and PP. As a result, the RC content in the PP matrix, which played a crystalline nucleation role, was reduced. During the crystallization process here, PP molecular chains were not arranged in a regular manner, such that the crystallinity of PP/RC composites decreased, which ultimately led to a reduction in the tensile modulus of the material.

The impact strength of these composites decreased with RC content (Figure 2). The impact strength of the composite was 10.88 kJ/m^2^ at 40 wt% RC content, which was 49% lower than that at 20 wt% RC content, mainly due to three reasons. First, RC addition destroyed the continuity of the resin matrix, which was not conducive to energy transfer and diffusion. Second, the RC rigid particles, as a dispersed phase, produced stress concentrations and were susceptible to the silver craze phenomenon when subjected to external forces, which led to defects within the composite. Third, due to RC’s low density, as the RC content increased, the proportion of volume occupied by RC increased and agglomeration increased, weakening bonding at the carbon/plastic interfaces.

### 3.2. Analysis of the Moisture Resistance of RC/PP Composites

The exposure of RC/plastic composite materials to moisture changes their dimensional stability properties, which also affects their antibacterial properties and mechanical properties. This study analyzed this aspect to provide a basis for the future production and application of RC/plastic composites.

**Figure 3:**
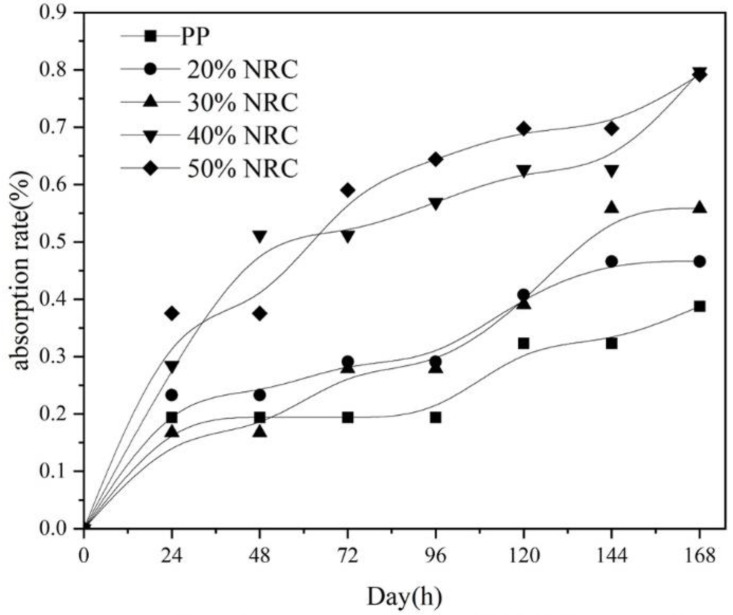
Moisture absorption of RC/PP Composites.

The relationship between water absorption of these composites over time for different RC contents showed that RC introduction slightly increased the moisture absorption of RC/PP composites (Figure 3). The moisture resistance of the composites slightly decreased, which was due to higher moisture absorption by RC. Because the RC exposed at the surface absorbed water and swelled, weak interfacial bonds between RC particles and the PP matrix led to an increased number of micropores, both of which were responsible for water absorption by the material [32,33]. However, the increased moisture absorption of RC/PP composites was not significant. Murayama et al. [34] studied the preparation of wood-plastic composites by adding wood flour to polypropylene, and the results showed that the composite reached a water absorption rate of 3.5% at 168 h. Yet, the RC/PP composite material had a low moisture absorption rate, all < 1%, as RC was well dispersed in the PP matrix. Meanwhile, the surface was better covered by the PP matrix, with the two forming a tighter structure and RC less likely to come into contact with moisture in the air, such that the RC/PP composite maintained better moisture resistance. The low water content of these RC/PP composites did not have the necessary substrate and environment for microorganisms to survive, thus achieving an antimicrobial effect, so RC/PP composites can be widely used in interior decoration, craft making, furniture, and other fields.

### 3.3. Thermal Weight Loss Analysis of RC/PP Composites

**Figure 4:**
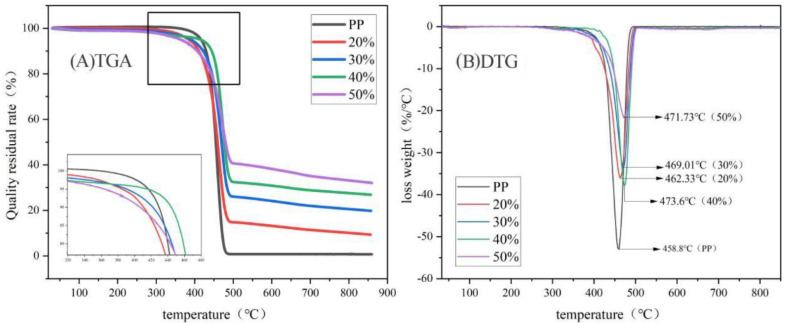
TGA curve and DTG curve of RC/PP Composites.

The results of thermogravimetric and differential thermogravimetric analyses (TGA and DTG, respectively) of these composites showed that these RC/plastic composites had no weight loss peaks between 0 and 200 °C, indicating that the composites had low water content (Figure 4 and Table 1). The starting decomposition temperature of PP was 416.5 °C, the fastest decomposition rate was 458.8 °C, the residual C rate at 550 °C was 0.73%, and it had almost no solid carbide generation [35] and no C formation.

Increased RC proportions reduced the onset of composite decomposition, resulting in increased decomposition temperatures, probably due to decomposition of volatile RC components. The results showed that there was only one decomposition temperature band for these RC/plastic composites (Figure 4). The initial decomposition temperature composites tended to increase and then decrease with increased RC content. The lowest onset decomposition temperature for RC/PP composites containing 50 wt% RC was 363.9 °C. The 40 wt% RC/PP composite exhibited better thermal stability, probably because, with RC powder addition, the migration of internal PP molecular chains was weakened and there was greater resistance to flow with heating, such that more thermal energy was consumed, thus enhancing composite thermal stability. As the RC content increased, the T_2_ value of RC/PP composites shifted to the right, indicating that the composite thermal stability increased, resulting in the ranking of the composites in terms of the residual RC content at 850 °C to the order of 50% > 40% > 30% > 20% > PP, which indicated that the residual RC rate of the composite increased with the RC content and was a result that has also been reported in other literature [36,37]. These composites were seen to be thermally stable with an appropriate RC content reinforced with PP, which had a certain retarding effect on the burning of the material.

### 3.4. Analysis of the Thermal Properties of RC/PP Composites (DSC)

The thermodynamic and thermal histories of these materials during processing were examined by the first temperature rise of the DSC. In experiments, analysis of the first temperature rise curve allowed the influence of the processing on the thermodynamic history of the material to be examined and discussed. Thus, the thermal history and influence of the thermodynamic history on the intrinsic structure of RC/PP composites were deduced from these experiments.

Formula for calculating the crystallinity of composite materials [26]:

*Xc* = (Δ*Hm*/Δ*Hm*^0^) × 100% (3)

where *Xc* is the composite crystallinity, %; Δ*Hm* is the composite melt enthalpy, J/g; and Δ*Hm*^0^ is the standard enthalpy of dissolution of pure PP at 100% crystallization, J/g; A review of the literature reveals that [26] Δ*Hm*^0^ = 209 J/g.

The T_onset_ (initial melting temperature) of pure PP was 152.26 °C and *X_c_*% (sample crystallinity) was 38.25%. When RC was added, the melt peak temperature of RC/PP composites did not significantly change compared to pure PP, while the starting melt temperature increased with RC content (Table 2). When 50 wt% RC was added, the starting melting temperature reached 155.76 °C, indicating that RC addition increased the heat resistance of these composites. Composite crystallinity data showed a curve that first increased and then decreased with RC. Although increased crystallinity can enhance the rigidity and hardness of the material [38], the heterogeneous nucleation of RC in the polymer should make the mechanical properties and heat resistance of the material more desirable. The mechanical properties of RC/PP composites with 40% RC content did not decrease with decreased composite crystallinity and the reasons for these analytical results might have been due to interfacial interactions between RC and PP. The mechanical properties of RC raw material, being an inherently brittle material, had a greater effect (as can be also seen in SEM). When 50 wt% RC was added, the RC/PP composite crystallinity was only 20.75%, which might have been due to the fact that RC addition restricted the movement of PP molecular chains and reduced their regularity and proportion of heterogeneous nucleation, thus reducing composite crystallinity [39].

### 3.5. Micromorphological Analysis of RC/PP Composites (SEM)

**Figure 5:**
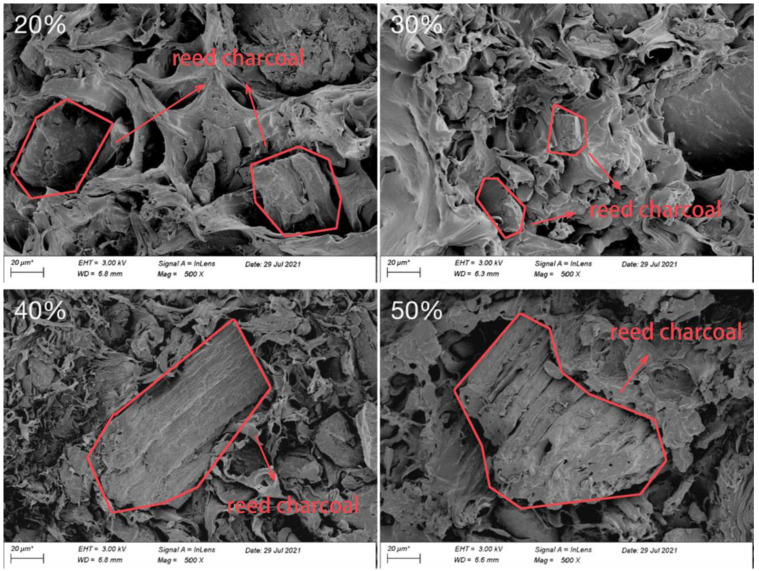
SEM images of tensile fracture surface of RC/PP Composites.

After tensile fracture, a sample was dried, sprayed with gold, and the section morphologies observed, which revealed the morphology of RC/PP composites with different RC/plastic ratios (Figure 5). When the RC content was 20 wt%, large areas of PP were observed after stretching. When external stress was applied, the PP matrix played a dominant role due to the low RC content. However, the PP macromolecular chains had become shorter, interaction forces between the molecular chains weakened, and composite mechanical strength decreased. When the RC content was 30 and 40 wt%, PP and RC were seen to be more evenly distributed and more tightly bonded. This indicated that PP molecules were well diffused into the RC surface pores before stretching, thus forming a strong physical interlock and stronger bonds between the two. When the RC content was 50 wt%, the matrix surface had RC particles exposed in brittle fractures, indicating that, at this time, when external stress was applied, RC played a dominant role. The structure did not immediately realize stress transfer within the body, resulting in stress concentration [40], and causing reduced composite tensile properties, such that excessive RC addition led to the reduction in the material’s two-phase compatibility.

### 3.6. Analysis of Dynamic Thermomechanical Properties of RC/PP Composites (DMA)

As a composite material consisting of polymeric materials, the properties of RC/PP composites are supposed to lie between those of an ideal solid and ideal liquid, i.e., they exhibit viscoelasticity. Therefore, the thermomechanical properties of RC/PP composites were characterized and analyzed using DMA. The test mode was set to a single cantilever mode with a temperature range from −15 to 180 °C, temperature rise rate of 3 °C/min, and frequency of 1 Hz. Energy storage modulus (E′), loss modulus (E″), and loss factor (δ) curves were obtained for the different RC/PP composites, using the relationship between the three, expressed as

(4) $$\mathrm{Tan}\delta =\frac{E^{\prime \prime }}{E^{\prime }}$$

where E″ is the loss modulus, which characterizes energy transformation into heat when a material is deformed, with a smaller value indicating a greater material stiffness; E′ is the energy storage modulus, also known as elastic modulus, which reflects material stiffness; and tanδ is a description of the material’s internal friction characteristics.

**Figure 6:**
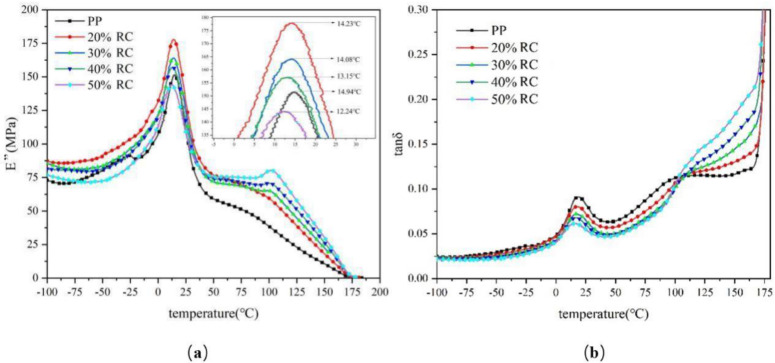
Loss modulus (**a**) and loss factor (**b**) curves of RC/PP composites at different temperatures.

**Figure 7:**
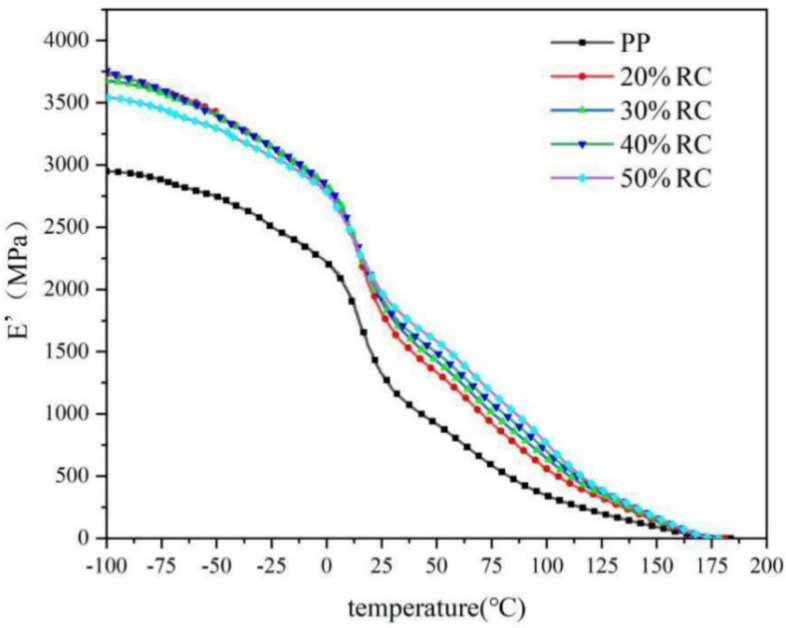
Storage modulus curve of RC/PP Composites.

(1) Modulus of loss (E″): This loss modulus can represent the energy loss in a material’s deformation or indicate its viscous behavior. The temperature spectrum of the material’s loss modulus showed that the loss modulus was greatest at 20 wt% RC in the composite (Figure 6a). According to ASTM E1640, the peak of the loss modulus temperature spectrum or the peak temperature of the loss factor temperature spectrum in the DMA data can be used to determine the glass transition temperature (Tg) of the material. The addition of charcoal powder was seen to reduce the composite glass transition temperature, such that the specimen glass transition temperature decreased with the charcoal content [41]. The observed decrease in glass transition temperature might have been related to the presence of pores in the composites.
(2) Loss factor (tanδ): The loss factor is the tangent of the angle between the strain and stress phase difference. Its value is equal to the ratio of the loss modulus to the energy storage modulus and reflects the viscous properties of the material. The higher the tanδ is, the greater the material’s internal energy dissipation, which corresponds to poor internal bond strength of the material and vice versa. In a certain temperature range, the tanδ of these composite materials increased with temperature, because thermal movement in the material was more intense at higher temperatures. The sliding ability of polymer chains thus increased, which was manifested as an increase in material flexibility. The loss factor curve of RC/PP composite specimens was examined as a function of temperature (Figure 6b). Below the glass transition temperature, the molecular chains in a polymer structure were frozen, such that all small groups and molecular chains hardly moved and, therefore, the polymer had a small damping factor and small loss factor. Thus, the loss factor of RC/PP composite specimens was very close to that of PP. However, when the test temperature was above the glass transition temperature, the loss factor of pure PP specimens increased more rapidly and was greater than that of RC/PP composite specimens. Above the glass transition of 100 °C, as the temperature increased, the molecular movement of PP intensified, thus increasing damping, while the movement of the PP molecular chains in RC/PP composite specimens was hindered by RC [42]. Comparing the five curves, as the RC content increased, composite tanδ values decreased, indicating that the corresponding composite had less internal energy consumption and that the internal bond strength of the material was higher than that of pure PP specimens.

(3) Energy storage modulus E′: The modulus of energy storage is used to describe the elastic aspect of a viscoelastic material, similar to the elastic modulus, which shows the ability of a material to resist deformation and store energy. The higher the energy storage modulus is, the greater the stiffness and greater the resistance to deformation. The temperature versus energy storage modulus for RC/PP composite specimens showed that, for specimens with different RC contents, the energy storage modulus increased with the RC mass fraction and then decreased (Figure 7). A maximum value in the glassy region was reached when the RC mass fraction reached 40 wt%. Within a certain range, the higher the RC mass fraction was, the higher the modulus of elasticity of composite specimens, which was consistent with the trend of increasing energy storage modulus with increased RC. Apparently, RC filled in the PP matrix to produce a good, rigid material and, when the composite was subjected to external forces, the RC and PP matrix shared the external load during material deformation and played good supporting roles before the glass transition. In contrast, RC occupied the space between PP molecular chains, restricting the movement of PP matrix molecular chains and hindering deformation of the PP matrix, thus increasing composite stiffness. The results obtained under DMA test conditions were that, below Tg, the E′ value did not change much and only slightly decreased. Then, around Tg, the E′ value sharply dropped, which indicated that the amorphous part of the material was in a transition between the glass and rubber states (i.e., the glass-transition region).

## 4. Conclusions

In this study, RC/plastic composites were prepared by melt-mixing and molding with carbonized reed as the main raw RC source. The effects of RC mass fraction on the physical and mechanical properties of these composites were examined. RC introduction had a good enhancing effect on the elastic modulus of these RC/PP composites, increasing composite stiffness, thereby reducing the impact properties of the PP matrix. The tensile and flexural elastic moduli of these composites were relatively good with 40 wt% RC, at 679.4 and 1862.8 MPa, respectively, which were 20.1 and 74.5% improved compared to those of composites with 20 wt% RC, respectively. In contrast, excess RC caused a continuous decrease in composite strength. These composites had good moisture resistance, with moisture absorption rates all below 1%. The starting decomposition temperature of RC/PP composites increased with the RC content and then decreased, with the highest starting decomposition temperature at 415.7 °C with 40 wt% RC, which was slightly lower than that of pure PP, and with the highest T_2_ value at 473.6 °C. The thermal stability of the 40 wt% RC/PP composite was the best. RC addition increased the starting melt temperature of these composites, with no significant change in the melt peak. At 50 wt% RC, the composite starting melting temperature reached 155.76 °C. The composite crystallinities showed a curve of increasing and then decreasing crystallinity with increased RC material and, with 50 wt% RC, the crystallinity was only 20.75%. By comparing the composite mechanical properties, the variation in thermal stability was observed, which showed more than the interactions between RC and PP. Crystallinity was also one of the main factors affecting the above-mentioned properties. A suitable RC/plastic ratio enabled PP molecules to diffuse well into RC surface pores, which resulted in a tighter bond between PP and RC, thus forming a strong physical interlock and stronger bonds between the two, while excessive RC proportions resulted in agglomeration when RC was not evenly dispersed in the PP matrix. Analysis of the dynamic thermo-mechanical properties showed that the material had the highest energy storage modulus at 40% RC, with a maximum of 3752.8 MPa, and showing a high degree of rigidity. At ~175 °C material, all composites melted, with the temperature of the tanδ gradually increased and material flexibility increased. The addition of RC powder reduced the composite glass transition temperature and, with increased RC content, the glass transition temperature of composite specimens decreased.

## Figures and Tables

**Table 1:** Thermal weightlessness of PP and RC/PP composites.

Sample	Starting Decomposition Temperature	Peak	850 °C Carbon Residue Rate
	T1	T2	Ash at 850 °C
	(°C)	(°C)	(%)
PP	416.5	458.8	0.73
20% RC	384.8	462.3	9.34
30% RC	390.6	469.0	19.80
40% RC	415.7	473.6	26.88
50% RC	363.9	471.3	32.03

Note: T1 is the temperature at which the material loses 5% of its weight and is defined as the onset of decomposition; T2 is the peak temperature of the DTG curve for the carbon plastic composite.

**Table 2:** Thermal behaviors of PP and RC/PP composites.

Sample	Sample Melting Parameters			
	Starting Melting Temperature	Melt Peak	Enthalpy of Melt	Crystallinity
	Tonset	*T_m_*	△*H_m_*	*X_c_*%
	(°C)	(°C)	(J/g)	
PP	152.26	164.63	79.96	38.25
20% RC	153.77	163.98	56.65	27.10
30% RC	153.96	163.88	70.04	33.51
40% RC	154.72	163.75	56.83	27.19
50% RC	155.76	163.73	43.37	20.75

## Data Availability

Not Applicable.
